# Thoracoscopic removal of dental prosthesis impacted in the upper thoracic esophagus

**DOI:** 10.1186/1749-7922-9-5

**Published:** 2014-01-14

**Authors:** Luigi Bonavina, Alberto Aiolfi, Stefano Siboni, Emanuele Rausa

**Affiliations:** 1 Department of Biomedical Sciences for Health, Division of General Surgery, University of Milano Medical School, IRCCS Policlinico San Donato, Via Morandi 30, 20097, San Donato Milanese, (Milano), Italy

**Keywords:** Esophagus, Esophageal perforation, Dental prosthesis, Thoracoscopy

## Abstract

Dental appliances are the most common cause of accidental foreign body esophageal impaction, especially in the elderly population with decreased oral sensory perception. A 47-year-old man with history of oligophrenia and recurrent epileptic seizures was referred to our hospital following dislocation and ingestion of his upper dental prosthesis. Endoscopic removal and clipping of an esophageal tear had been unsuccessfully attempted. A chest CT scan confirmed entrapment of the dental prosthesis in the upper thoracic esophagus, the presence of pneumomediastinum, and the close proximity of one of the metal clasps of the prosthesis to the left subclavian artery. A video-assisted right thoracoscopy in the left lateral decubitus position was performed and the foreign body was successfully removed. The patient was then allowed to wear the retrieved prosthesis after dentistry consultation and repair of the wire clasps by a dental technician. At the 6-month follow-up visit the patient was doing very well without any trouble in swallowing.

## Introduction

Accidental ingestion of foreign bodies is frequent in adult individuals with mental retardation or psychiatric disorders. Most of the little ingested foreign bodies pass the gastrointestinal tract without consequences. However, 10-20% of the patients may require endoscopic removal, and 1% or less may require surgery due to entrapment of the foreign body in the cervical (57%), thoracic (26%), or distal (17%) esophagus [1]. Dental appliances are the most common cause of accidental foreign body esophageal impaction, especially in the elderly population with decreased oral sensory perception [2]. The large size, sharp edges, and metal clasps of dental prostheses make endoscopic removal unsafe and carry a high risk of perforation in such circumstances. We present a case of successful thoracoscopic removal of dental prosthesis impacted in the upper thoracic esophagus.

## Case report

**Figure 1:**
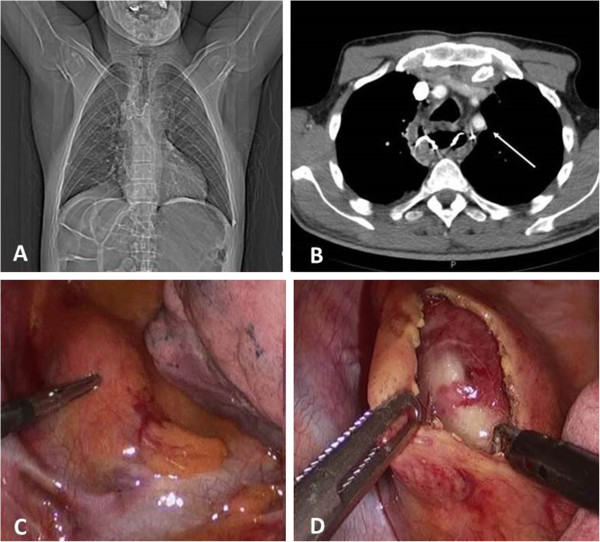
Appearance of the dental prosthesis at CT scan (A-B), and thoracoscopic exposure of the upper thoracic esophagus (C-D).

A 47-year-old man with history of oligophrenia and recurrent epileptic seizures was referred to our hospital 3 days after dislocation and ingestion of his upper dental prosthesis. Before patient’s referral, multiple flexible endoscopic attempts had been unsuccessfully performed, the last one leading to an intramural perforation partially repaired with endoclips. The patient’s main complaints were dysphagia, odynophagia, and hypersalivation. He was afebrile, with normal leucocyte count, and slight elevation of C-reactive protein. Broad-spectrum antibiotic therapy (piperacillin + tazobactam) was started upon hospital admission. The physical examination did not reveal subcutaneous emphysema. A gastrografin swallow study showed extravasation of contrast at the level of the upper thoracic esophagus; a chest CT scan confirmed the presence of pneumomediastinum and the close proximity of one of the metal clasps of the prosthesis to the left subclavian artery (Figure 1A-B).

**Figure 2:**
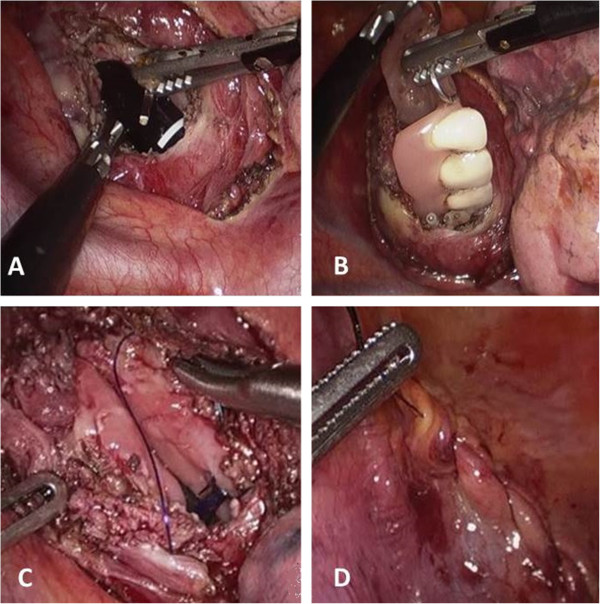
Esophagotomy (A), removal of the dental prosthesis (B), and suture of the esophageal wall and mediastinal pleura (C-D).

A video-assisted right thoracoscopy in the left lateral decubitus position was performed to remove the foreign body. Three ports were used: 10 mm optical port in the 6^th^ intercostal space, 10 mm port in the 5^th^ intercostal space, and 5 mm port in the 4^th^ intercostal space, and. The exploration of the right chest showed a bulging of the upper mediastinal compartment above the confluence of the azygos vein into the superior vena cava (Figure 1C). There was no pleural contamination. After incision of the thickened mediastinal pleura (Figure 1 D), transillumination with a standard endoscope confirmed the site of impaction and the previous perforation. The esophagus was opened longitudinally for approximately 4 cm and the prosthesis (five dental elements with three metal clasps) was removed under direct endoscopic and thoracoscopic view using an endograsper (Figure 2A-B), and enveloped in a plastic bag. The edges of the esophagomyotomy appeared vital. The esophageal wound was closed with a double-layer running suture of Polydioxanone 3–0 including the mucosa and the muscle layers, and tested for air-leakage (Figure 2C-D). The mediastinal pleura was then approximated with a running suture. The plastic bag containing the dental prosthesis was removed from the anterior trocar site by slightly enlarging the incision. The postoperative course was uneventful. A gastrographin swallow study performed on postoperative day 3 showed a regular esophageal transit and the absence of leaks. The patient was then allowed to wear the retrieved prosthesis after repair of the wire clasps by a dental technician and dentistry consultation. He was discharged well from the hospital on postoperative day 8 on a free diet. At the 6-month follow-up visit the patient was doing very well without any complaint in swallowing.

## Discussion

The frequency of removable dental prostheses among adults varies between 13 and 29% in Europe, with 3-13% of edentulous subjects wearing complete dentures in both jaws; interestingly, there is a trend towards an increasing use of removable partial dentures [3]. It is therefore reasonable to estimate that, with the growth of the denture-wearing population, the incidence of impacted dentures in the esophagus may increase in the future. Impacted dental prostheses in the esophagus can result in life-threatening conditions such as mediastinitis, pleural empyema, and aortoesophageal fistula [4]. The risk of severe complications is higher in patients with a delayed diagnosis and treatment, since long-standing impaction can lead to mucosal ulceration, transmural inflammation, esophageal perforation, and sepsis.

The diagnosis of denture impaction in the esophagus may be challenging in patients with mental disorders who may be unable to give a reliable medical history. Since dentures are made of acrylic resin, which is radiolucent, the patient work-up should routinely include a chest X-ray, a gastrografin swallow study, a computed tomography, and an upper endoscopy. These investigations are essential to define the anatomical site of impaction and the size, shape, and number of wire clasps of the prosthesis.

Attempts at endoscopic removal of the dental prosthesis may cause intramural perforation or a full-thickness tear due to the possible entrapment of the wire hooks in the esophageal wall. Esophagotomy through a right thoracotomy remains the safest therapeutic approach when the impaction occurs in the upper thoracic esophagus. Video-assisted thoracoscopy, either in the left lateral or prone decubitus position, allows a safe and minimally invasive retrieval of the dental prosthesis followed by primary esophageal suture when there is no major pleural contamination and the edges of the esophagomyotomy appear vital. In the literature, a few cases of thoracoscopic removal of ingested foreign bodies have been reported; three of the 6 patients required an esophagotomy due to an impacted denture (Table 1). In our patient, thoracoscopic removal was successfully performed after previous failed endoscopic procedures complicated by intramural perforation. Exposure of the upper thoracic esophagus was possible without the need to divide the arch of the azygos vein.

**Table 1:** Thoracoscopic management of ingested esophageal foreign bodies in adults: literature review

**Author**	**Year**	**Description**	**Surgical approach**	**Operative decubitus**	**Treatment**	**Outcome**
Davies B. [5]	2004	China cup fragment migrated in the mediastinum, with abscess	Right-side thoracoscopy (3-port access)	NS	Foreign body removal and abscess drainage	Good
Palanivelu C. [6]	2008	Impacted denture	Right-side thoracoscopy (3-port access)	Prone	Esophagotomy, foreign body removal and suture	Good
Rückbeil O. [7]	2009	Metallic needle migrated in the mediastinum	Right-side thoracoscopy (3- port access)	Left lateral	Foreign body removal	Good
Dalvi AN. [8]	2010	Impacted denture	Right-side thoracoscopy (4-port access)	Left lateral	Esophagotomy, foreign body removal and suture	Good
Fujino K. [9]	2012	Fish bone migrated to lung	Right-side thoracoscopy (NS)	NS	Foreign body removal	Good
Present case	2013	Impacted denture	Right-side thoracoscopy (3-port access)	Left lateral	Esophagotomy, foreign body removal and suture	Good

(NS: non specified).

Based on our experience and the available literature we conclude that thoracoscopic esophagotomy represents a safe and effective treatment for patients with impacted dentures in the esophagus. Multiple attempts at flexible and rigid esophagoscopy should definitely be abandoned in such patients, especially when a dental prosthesis has passed the cricophageal sphincter. Education and close follow-up of patients wearing removable dental prostheses is critical to prevent accidental impaction in the esophagus and the dangerous sequelae of esophageal perforation.

## Competing interests

The authors declare that they have no competing interests.

## Authors’ contributions

LB designed and wrote the manuscript, AA, SS, and ER contributed to data collection and manuscript drafting. All authors read and approved the final manuscript.
